# Generalized resistance to pruritogen-induced scratching in the C3H/HeJ strain

**DOI:** 10.3389/fnmol.2022.934564

**Published:** 2022-10-05

**Authors:** Yanbin Zhang, Nicole Richter, Christine König, Andreas E. Kremer, Katharina Zimmermann

**Affiliations:** ^1^Department of Anesthesiology, Friedrich-Alexander-University Erlangen-Nürnberg, Erlangen, Germany; ^2^Department of Gastroenterology and Hepatology, University Hospital Zürich, Zurich, Switzerland; ^3^Department of Medicine 1, Friedrich-Alexander-University Erlangen-Nürnberg, Erlangen, Germany

**Keywords:** pruritus, scratching behavior, itch, inbred mouse strains, C57BL/6J, heritability

## Abstract

Previously the effect of the pruritogens, such as histamine and chloroquine, was tested in 11 inbred mouse strains, and this study aimed to identify resistant and sensitive strains, consistent with the observation that underlies the large variability in human populations. In the present study, we used the low responder C3H/HeJ (C3H) and the more sensitive C57BL/6J (C57) strain to find out if resistance and sensitivity to develop pruritus is restricted to only histamine and chloroquine or extends to other known pruritogens as well. We tested five additional commonly known pruritogens. We established dose-response relationships by injecting four concentrations of the pruritogens in the range of 0.3, 1, 3, and ten-fold in the nuchal fold. Then we assessed the scratching behavior for 30 min after injection with an automated custom-designed device based on the bilateral implantation of mini-magnets in the hind paws and on single cages placed within a magnetic coil. We found that the resistance to pruritogens is a general phenotype of the C3H strain and extends to all pruritogens tested, including not only histamine and chloroquine, but also endothelin, trypsin, 5-HT (serotonin), the short peptide SLIGRL, and Lysophosphatidic acid (LPA). C57 was more sensitive to all pruritogens and, in contrast to C3H, dose-response relationships were evident for some of the pruritogens. In general, comparable peak scratch responses were observed for the 0.3-fold concentrations of the pruritogens in C57 whereas C3H required at least the ten-fold concentration and still displayed only between 5 and 33% of the scratch responses observed in C57 for the respective pruritogen. The general resistance to pruritogens and the low level of scratching behavior found in the C3H strain is an interesting trait and represents a model for the study of the heritability of itch. It is accompanied in C3H with a higher sensitivity in assays of nociception.

## Highlights

- A previously recognized resistance to developing scratching in response to histamine and chloroquine in the C3H/HeJ mouse strain extends to other commonly known pruritogens.
- C57BL/6J mice show several fold higher sensitivities to all pruritogens tested.
- The opposite phenotype observed in these inbred strains may provide an interesting and translational model for the study of genetic factors for itch and is representative of other inbred mouse strains.

## Introduction

“Itch” can be explained as an unpleasant sensation on the skin that is followed by the urge to scratch. From an evolutionary point of view, acute pruritus is a protective alarm signal to defend the skin from harmful damage. The reflex-induced scratching is supposed to remove the potentially dangerous stimulant and bring relief by activating the reward system in the brain (Mochizuki et al., 2014).

Short-lasting pruritus is triggered by numerous agents acting via two main receptor families: G protein-coupled receptors, such as the family of mas-related G-protein coupled receptors and transient receptor potential channels, which are located on unmyelinated type C and thinly myelinated type Aδ nerve fibers in the skin. Until the late 1990s, itch was hypothesized to be a subthreshold activation of nociceptors, which signal pain and share common pathways (intensity theory). Only in 1997, a specific itch-related pathway was identified (Schmelz et al., 1997). Currently, the overabundance of new information on pathways and mediators for itch has led to the belief that transmission occurs via dedicated sensory-labeled lines which are identified by the respective marker molecules and projected in a complex network of itch- and pain-related neurons in the spinal cord. This view competes with the pattern theory of itch according to which nociceptors signal itch or pain, depending on either the combination of activated fibers or characteristic discharge patterns (Schmelz, 2021).

When pruritus becomes chronic, that is, persists for more than 6 weeks, its etiology becomes complex, for example, in the context of dermatological diseases, clinical neuropathy, and chronic diseases of inner organs including the liver and kidney (Mettang and Kremer, 2015; Hussain et al., 2019; Steinhoff et al., 2019; Kahremany et al., 2021). Therapeutics, that specifically target chronic itch, are currently not available. Nevertheless, a few antipruritic drugs which target specific receptors in the nervous system are meanwhile accessible (Fowler and Yosipovitch, 2020; Sutaria et al., 2022). According to the Global Burden of Disease study, pruritus is among the 50 most common causes of disease (Hay et al., 2014) and it is estimated that ~20%−27% of the world's population suffer from chronic pruritus at least one time in their lifetime which underlines the clinical relevance of the problem and the need for targeted therapeutic strategies (Weisshaar, 2016).

No matter which of the transmission theories for itch proves ultimately true, itch, like pain (Fillingim, 2017), exhibits large interindividual variability, as observed with human subjects and atopic dermatitis patients: in this study, individual differences in skin pathophysiology and psychological state were suggested as possible reasons for the observed differences (Solinski and Rukwied, 2020). Further examples are provided based on the side effects of specific drugs. After systemic treatment with opioids, for example, pruritus occurs in about 2%−10% of patients. The risk is markedly increased when opioids are given epidurally or intraspinally, and the highest prevalence (up to 100%) is associated with intrathecal morphine (Reich and Szepietowski, 2010). Similarly, the anti-malarial drug, chloroquine phosphate, is associated with intolerable itch, which is seen as a side effect in 8%−20% of patients with malaria. The given incidence refers to data obtained from different community hospitals in Nigeria, depending on the respective incidence of clinical malaria (Sowunmi et al., 1989). Usually, pruritus is severe and incapacitating. It develops within 6–8 h of taking the drug and is unresponsive to anti-histamines and remits spontaneously within 48–72 h. It affects the hands, feet, and scalp, including the mucous membranes of the eyes, mouth, and genitalia are remarkably free, likewise, there is variability in pruritus observed in patients with malaria treated with halofantrine, however, usually patients with sensitivity to halofantrine-induced pruritus also reported a personal or family history of itching on chloroquine (Sowunmi et al., 1989; Ezeamuzie et al., 1991). Beyond these observations, there is evidence that genetic predisposition, that is, heritability, is an important factor in mediating variable sensitivity to pruritus. In this context, up to 74% of dark-skinned Africans experience severe itching after coming in contact with chloroquine, with little incidence observed among Asians and Europeans. The number of such cases were assessed by a cross-sectional epidemiological survey amongst a population of 20,000 at the Obafemi Awolowo University campus in Nigeria (Ajayi et al., 1989; Tey and Yosipovich, 2010).

A previous study by Green et. al. in 11 inbred mouse strains, induced histamine- and non-histamine-dependent pruritus by injecting histamine and chloroquine in the nape of the neck and reported a large influence of the genetic background on the intensity of the scratch response. The authors of the mentioned study also observed differences between the sexes with female mice scratching 23% more than male mice. Thus, in mice and similar to nociception, heritable factors underlie for the cause of pruritus (Mogil et al., 1999, 2005; Green et al., 2006; Mogil, 2012).

In the study by Green et al., C3H/HeJ (C3H) was identified to be one of the inbred strains with little sensitivity to chloroquine and histamine. Here we tested if the low sensitivity to chloroquine and histamine observed in C3H extends to other commonly known pruritogens, including endothelin, trypsin, 5-HT (serotonin), SLIGRL, and Lysophosphatidic acid LPA, (Zelenin et al., 2021). Therefore, in this study, we injected the pruritogens in the nape of the neck and compared the scratch responses to the more widely used C57BL/6J (C57) strain.

## Materials and methods

### Materials

For the injection of pruritogens, we used Omnican^®^ F 1 ml syringes with hypodermic needles (Braun, Kronberg IM Taunus, Germany). Before injection, all pruritogens were dissolved in phosphate-buffered saline (PBS) except LPA, which was dissolved in 0.1% mouse serum albumin (MSA) and PBS. All pruritogens and the respective stock concentrations are listed in Table 1.

**Table 1:** Stock concentration and provider information for the pruritogens.

**Pruritogen**	**Supplier**	**Stock concentration**	**Reference**
Endothelin	Enzo	100 μM	Imamachi et al. (2009), Liang et al. (2010)
Chloroquine	Resochin from Bayer Schering Pharma	400 mM	Akiyama et al. (2013)
Trypsin	Sigma Aldrich	1.01 kU/μl	Costa et al. (2008), Liu et al. (2011)
SLIGRL-NH2	Biomatik	200 mM	Imamachi et al. (2009)
Lysophosphatidic acid	Avanti Lipids	40 mM + 10% MSA in PBS	Hashimoto et al. (2004)
Serotonin/5-HT	Biomol	100 mM	Akiyama et al. (2013)
Histamine dichloride	Carl Roth	8.9 M	Han et al. (2006), Akiyama et al. (2012b)

### Animals

Breeding pairs of C57BL/6J (C57) and C3H/HeJ (C3H) strains were purchased from the Jackson Laboratories (Bar Harbor, Maine, USA) *via* Charles River, Germany (Stock Numbers 664 and 659). Colonies were established using brother × sister or offspring × parents mating. For C3H, 21 females and 30 males were used. In female mice, the age ranged between 57 and 112 days (71.42 ± 17.21 days) and weight between 15.5 and 21.4 g (18.33 ± 1.59 g). Whereas in male mice, age ranged between 57 and 112 days (79.8 ± 19.17 days), and weight between 20.7 and 28.9 g (26.64 ± 2.31 g). For the C57 mouse strain, 24 females and 30 males were used. In female mice, age ranged between 66 and 96 days (81.53 ± 9.31 days) and weight between 18.5 and 22.7 g (21.10 ± 0.98 g). Whereas in male mice, age ranged between 59 and 99 days (79.8 ± 13.10 days), and weight between 21.1 and 28.7 g (25.15 ± 2.02 g). The mice were housed at the Preclinical Research Center of the Universitätsklinikum Erlangen in Erlangen, Germany. Mice were kept in groups with a minimum of two and maximum of five animals in one cage, respectively, according to their litter size after birth and to FELASA recommendations. All mice were kept under a 12:12 h light: dark cycle regulated between 4:30 AM and 4:30 PM. Ambient temperature and humidity were kept at 22 ± 2°C and 55 ± 10%, respectively. Food and ozonized tap water were available *ad libitum*.

### Quantification of scratch behavior in mice

Scratching in mice is a characteristic and uniform movement with restriction to the hind paws. In order to automatically quantify this type of behavior, we used a magnetic coil-based detection system, where the mice get small magnetic implants in both hind paws. The magnets are placed under the skin and below the knee. For placement of implants, we anesthetized mice with isoflurane (2%−3%) using a face mask. Then the hind legs were shaved up to the hips with a razor (Aesculap, Tuttlingen). On this occasion, the mice were also shaved in the neck fold to prepare the skin for the pruritogen injection. For subcutaneous injection of magnets, both hind legs were held in position with tape and a 2 mm skin incision was made at the level of the hip on both sides. PTFE-coated magnets (size 5 × 2 mm, VWR International) were implanted subcutaneously in both hind legs with the help of a pet chip injector. The PTFE-coating prevents tissue response against the implants, and during the course of this and other studies, we never observed tissue inflammation due to foreign body rejection. The proper position of the magnet was verified manually at the end of each surgery and the resulting skin incision at the level of the hipbone was sewed with one stitch. Afterward, the mice placed back into its home cage. After at least 7 days of recovery in their home cages, the mice were transferred to the experimental room. They were single-housed during experiments and therefore placed in individual cages at least 120 min before the actual measurement in order to acclimate them to the measurement environment. They had free access to food and water, but no enrichment or nesting materials. For the measurements, the cages were placed in a frame with a magnetic coil. Within this frame, every movement of the hind limbs led to the movement of the magnet implants and induced an induction current in the magnetic field. This current was registered in gap-free mode and analyzed with custom-designed software. Immediately before the measurements, the mice were carefully taken out of their individual cages, the hindlimbs were manually checked for correct placement of magnets, and then the mice were released in a mouse restrainer. Then the pruritogens, diluted in PBS to the respective concentration, were injected with a hypodermic needle into the skin of the neck fold. Mice were gently released from the restrainer and put back in the measurement cages. From this moment onward, the acute scratching behavior was registered for 30 min. The pruritogens were tested per group and in each group, four concentrations were tested. The smallest concentration was injected on the last day, in order to become aware of potential sensitization. There were intervals of 48 h between the injection of the different concentrations. The pruritogens injection sequence and dosage are outlined in Table 2.

**Table 2:** Pruritogens and their respective dose and sequence of injection.

**Pruritogen**	**Day 1** **3-fold**	**Day 3** **1-fold**	**Day 5** **10-fold**	**Day 8** **0.3-fold**
Endothelin	373.78 ng	124.59 ng	1.24 μg	37.37 ng
Chloroquine	191.94 μg	63.98 μg	639.8 μg	19.19 μg
Trypsin	606 μg	202 μg	2.02 mg	60.6 μg
SLIGRL	197.34 μg	65.8 μg	657.85 μg	1.97 μg
LPA	261.9 μg	87.3 μg	873 μg	28.37 μg
5-HT	26.43 μg	8.81 μg	88.11 μg	2.64 μg
Histamine	1.48 mg	494.61 μg	4.94 mg	143.38 μg

### Data analysis and statistics

Scratch data were recorded using the custom-designed scratch intensity monitoring *SiMon* software (V2.0, Academic Medical Center, University of Amsterdam), and 30 min periods were extracted offline for every mouse individually using the *Scratch Analysis* software (V1.13, Academic Medical Center, University of Amsterdam). The resulting 30 min test files were then analyzed for scratch time, bouts, and events by a custom-made Python program written by Ron Castel. All data were then further processed in Excel (Version 2016) and plotted in R. From there, scratching was analyzed as scratch time (the total time of scratching in a 30 min test period), the number of scratch bouts (the number of uninterrupted scratch movements in a 30 min test period) and the number of scratch events, whereby scratch events contained several scratch bouts from the moment of one paw lift until the paw was let down. There was a maximal coefficient of variation of 40% between the beats of one bout (Kremer et al., 2010). Statistical analysis was performed using SPSS or R. Outliers were removed based on the 2.2-fold of the interquartile range (Hoaglin and Iglewicz, 1987). The scratch response to the seven pruritogens (histamine, chloroquine, 5-HT, SLIGRL, LPA, trypsin, and endothelin) was measured in the two strains. Male and female animals were pooled for the analysis as there were no apparent differences between the sexes. Nevertheless, we distinguished the sexes in the respective figures. After outlier analysis, the groups were subjected to the Kolmogorov–Smirnov normality test. A total of 47 out of 53 groups were normally distributed (88.67%). Statistical differences between groups and doses were analyzed using repeated measures analysis of variance (ANOVA) using R. The distinction between the groups was made based on *p* < 0.05. Data were presented as mean ± SEM, *p* < 0.05 was considered statistically significant.

## Results

**Figure 1:**
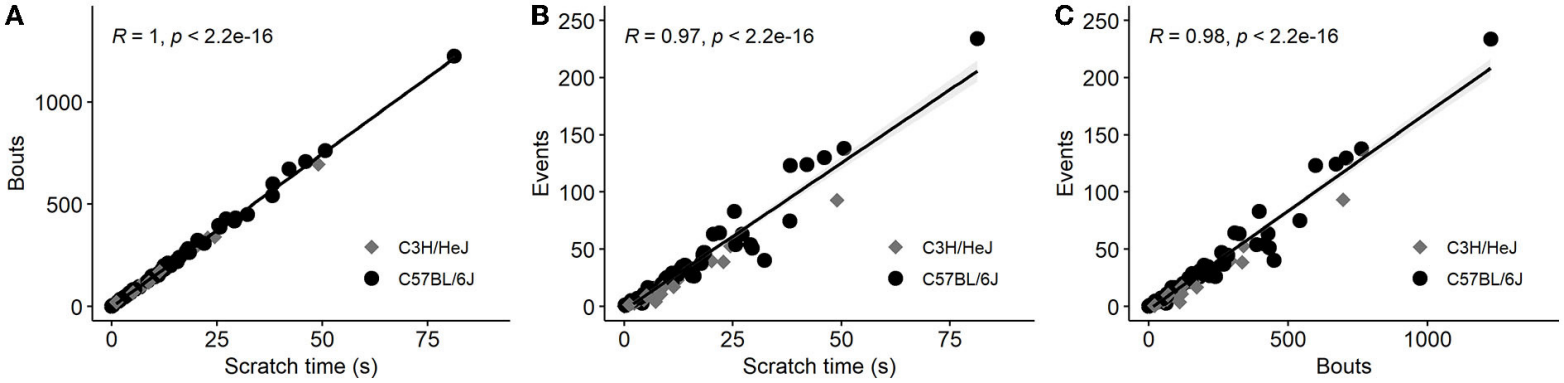
Pearson correlation matrix of scratching time, scratch bouts, and scratch events summarizing all data points from a one-fold concentration of each pruritogen in C3H and C57 mice. Pruritogens were injected into the nape of the neck, scratch behavior was measured immediately for 30 min and values were summed. Scratch time represents the cumulative time in s, scratch bouts were counted as the number of uninterrupted scratch movements in a 30 min test period and the scratch events consisted of several scratch bouts from the moment of one paw lift until the paw was let down. All three parameters were recorded independently by the software for every mouse and correlated with each other. **(A,B)** Illustrate the correlation of scratch time with scratch bouts and scratch events, and **(C)** the correlation of scratch bouts with scratch events. Each point represents data from one individual mouse. All parameters are highly correlated (*n* = 97).

**Figure 2:**
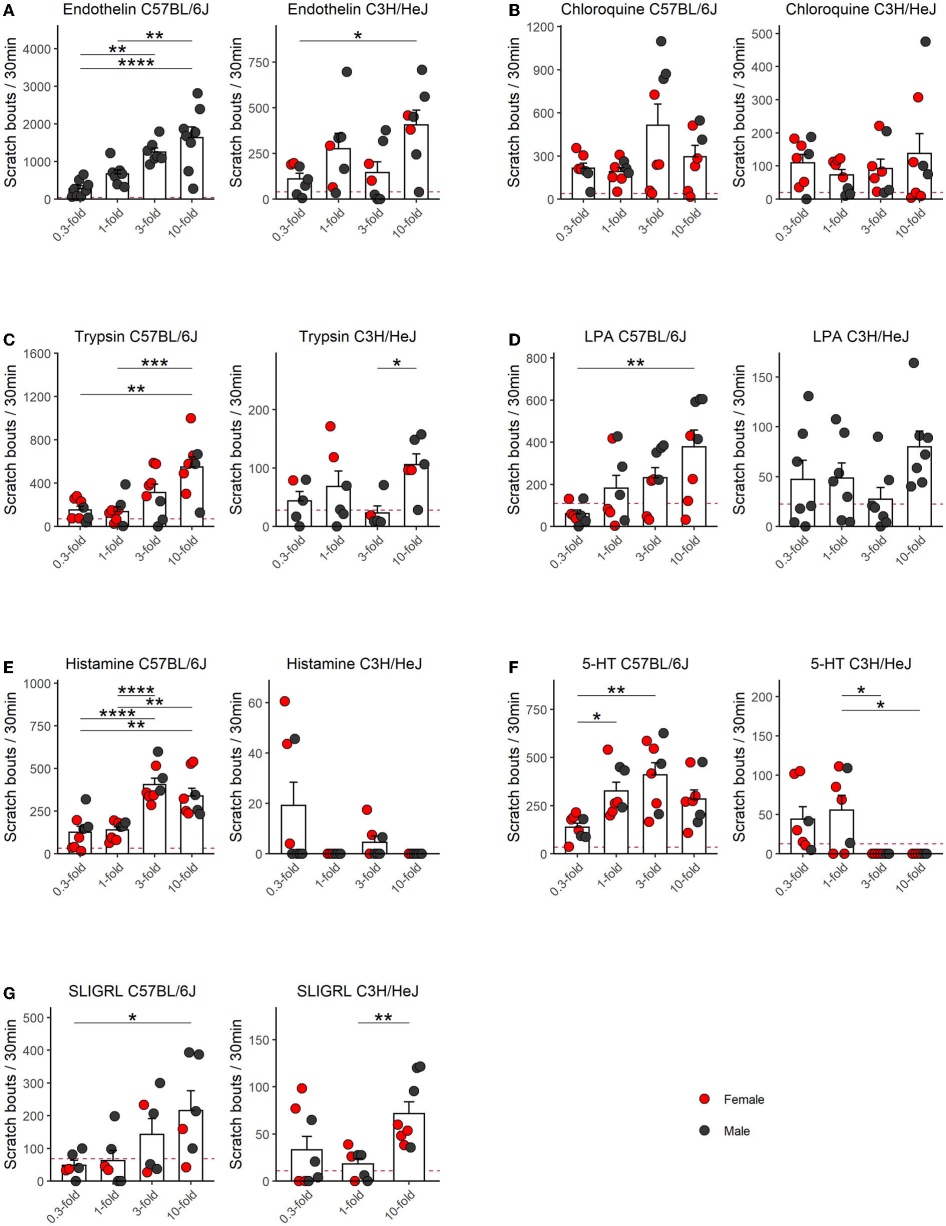
Dose–response relationships of scratching behavior in C57 and C3H mice for seven pruritogens. Columns represent means ± SEM of scratch bouts in 30 min following an injection of, **(A)** endothelin (0.3–10 μM; C57 *n* = 8 males; C3H *n* = 7, two females, five males), **(B)** chloroquine (1.2–40 mM; C57 *n* = 6, four females, two males; C3H *n* = 8, three females, five males), **(C)** trypsin (3.03–101 U/μl; C57 *n* = 8, five females, three males; C3H *n* = 6, two females, four males), **(D)** LPA (1.2–40 mM; C57 *n* = 8, four females, four males; C3H *n* = 7, seven males), **(E)** histamine (26.7–890 mM; C57 *n* = 6, four females, two males; C3H *n* = 8, three females, five males), **(F)** 5-HT (0.3–10 mM; C57 *n* = 8, five females, three males; C3H *n* = 7, five females, two males), **(G)** SLIGRL (0.6–20 mM; C57 *n* = 7, two females, four males; C3H *n* = 8, four females, four males) into the nape of the neck. The red dashed line indicates the mean response of an interpolated PBS control with the one-fold concentration of a different animal cohort (see Supplementary Figure 2). In C57, endothelin, trypsin, LPA, and SLIGRL, displayed linear dose-response relationships with the 10-fold concentration having the largest effect while in the remaining pruritogens, namely chloroquine, 5-HT, and histamine, we identified the doses with maximum effect at the three-fold concentrations. All pruritogens were several-fold more potent in C57 as compared to C3H. *p*-Values result from repeated measures ANOVA with Tukey's HSD *PostHoc*Test. Not normal distributed groups (Shapiro–Wilk test) were compared by using Wilcoxon rank-sum test. Asterisks indicate the level of significance: ****p ≤ 0.0001, ***p ≤ 0.001, **p ≤ 0.01, *p ≤ 0.05. Data points from females are red and males are black.

In order to quantify scratching behavior, our software recorded three different parameters, that is, scratch bouts, scratch events, and scratch time. We found a very high correlation between these parameters (Figure 1). As given in the literature, the number of scratch bouts has been most frequently used to quantify and visualize the scratch behavior. Therefore, we used mostly bouts for the analysis, but an analysis of scratch events is available in the Supplementary Figure 1. For each pruritogen, histamine, endothelin, SLIGRL, chloroquine, LPA, 5-HT, and trypsin, we included four concentrations and the data were displayed as an average of scratch bouts over 30 min. Shortly after the injection of the pruritogens, mice developed the scratch response. We observed that this scratch response, counted as scratch bouts, was observed for all pruritogens, at their most effective dose, with several folds larger in the C57BL/6J (C57) mouse strain than in the C3H/HeJ (C3H) mouse strain (Figure 2, see Supplementary Figure 1 for the respective scratch events).

Overall endothelin was the most potent pruritogen in both strains. In C57 it produced the largest number of bouts: at the highest concentration, ten-fold, these were 1,633 ± 289 bouts (*n* = 8 males) while the lowest dose produced 280 ± 77 bouts. The dose-response relationship was linear and increasing doses produced significantly larger responses (*F* = 13.8, *p* = 0.00005). In the C3H strain, the 10-fold concentration was also the most potent, but more than fourfold less effective. It provoked 405 ± 82 bouts (*n* = 7, two females, five males), with a smaller, but significant effect of dose (*F* = 5.3, *p* = 0.009). The 0.3-fold concentration produced the least number of bouts, which was 111 ± 30 (Figure 2A). In both strains, the endothelin-induced scratch bouts, were many folds higher than a previously conducted PBS control in a different population of C57 and C3H mice of both sexes and using one-fold of the respective pruritogen concentration (Supplementary Figure 2). The respective expected PBS control level is represented as the horizontal dashed line in Figure 2A for endothelin and in the subsequent panels relative to the respective other pruritogens.

In contrast to endothelin, the chloroquine dose-response relationship covered the maximum dose in C57. Here, the three-fold concentration was most potent (513 ± 146) and the one-fold concentration produced the smallest reaction (192 ± 28; *n* = 6, four females, two males). Previously an inverted U-shaped dose-response profile was described for chloroquine (Green et al., 2006), but we did not find a significant difference between the three- and ten-fold concentrations in our experiments. The effect of dose was significant (*F* = 3.2, *p* = 0.045). In C3H, the largest response occurred with the ten-fold dose (138 ± 59; *n* = 8, five females, three males) and the smallest as well with the one-fold dose (73 ± 17). In C3H, chloroquine produced a 3.7-fold smaller response than the most potent dose in C57 and we observed no relevant effect of dose (*F* = 0.8, *p* = 0.5; Figure 2B).

In C57, the doses in the respective concentration range for trypsin (*n* = 8, five females and three males) and LPA (*n* = 8, four females and four males) produced increasing dose-response relationships and scratch responses of almost similar extent (*F* = 8.4 and *p* = 0.0007 for trypsin and *F* = 8.1 and *p* = 0.0008 for LPA). For both pruritogens, the ten-fold concentration was most potent and resulted in 549 ± 92 and 378 ± 80 bouts, respectively. The lowest responses were provoked by one-fold (138 ± 43) and 0.3-fold (61 ± 17) bouts, respectively. In C3H, these two pruritogens produced at the most potent concentration five-fold smaller responses. For both pruritogens, the ten-fold concentrations were most potent and produced for trypsin 106 ± 19 (*n* = 6, two females and four males) and LPA 78 ± 16 bouts (*n* = 7 males), respectively (Figures 2C,D). Unlike endothelin, trypsin and LPA produced no dose-dependent effects in C3H (*F* = 2.7 and *p* = 0.09 for trypsin and *F* = 1.9 and *p* = 0.2 for LPA), but at least for trypsin, the ten-fold concentration produced significantly more scratch bouts than the three-fold concentration.

In C57, both histamine (*n* = 6, four females, two males) and 5-HT (*n* = 8, five females, three males) were equally effective and they produced both inverted U-shaped response-relationships with respect to scratch events (Supplementary Figure 1, *F* = 14.4 and *p* = 0.00005 for histamine and *F* = 7.5 and *p* = 0.001 for 5-HT), but with respect to scratch bouts, the highest concentrations were not significantly different (*F* = 15.7 and *p* = 0.00001 for histamine and *F* = 6.9 and *p* = 0.002 for 5-HT). The largest number of scratch bouts were provoked by the three-fold doses, and these were 406 ± 37 and 409 ± 63, respectively. In C3H, histamine was the least effective of all pruritogens and provoked the highest response at 0.3-fold concentration, which was 19 ± 9 bouts in 30 mins (*n* = 8, three females, five males). Differences between the doses were small, but significant (*F* = 3.7 and *p* = 0.04). Similarly, 5-HT produced very little response, at the most 55 ± 19 bouts at the one-fold concentration (*n* = 7, five females, two males), although the dose, according to the ANOVA, still had a significant effect (*F* = 5.5, and *p* = 0.008). Taken together, histamine and 5-HT were therefore at least 7–21-fold more potent pruritogens in the C57 strain and C3H was practically resistant (Figures 2E,F).

**Figure 3:**
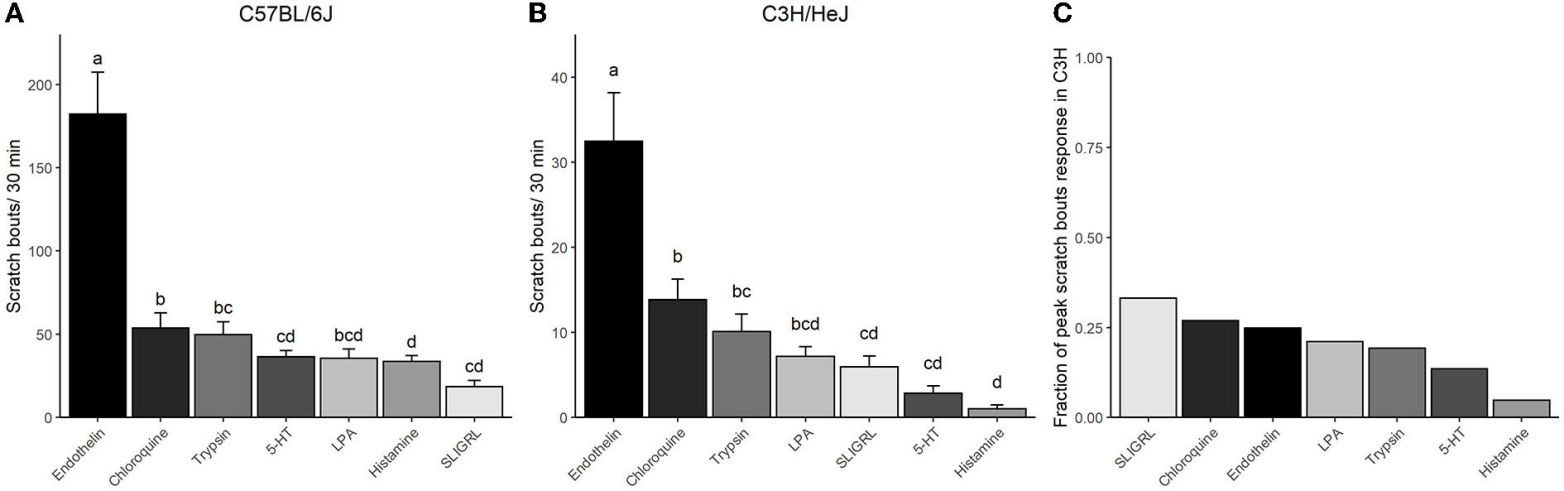
Comparison of the efficacy of the seven pruritogens in C57 and C3H. Columns represent means ± SEM scratch bouts for 30 min following injection of the respective pruritogens at four concentrations into the nape of the neck of **(A)** C57 and **(B)** C3H mice. **(A,B)** The scratch bout response to the respective pruritogens was averaged for the four concentrations and ranked from the largest to the smallest response. *p*-Values indicate the level of significance and result from a repeated measures ANOVA with Tukey's HSD *PostHoc*Test. **(C)** The bar chart expresses the relative highest scratch response observed in C3H as the fraction of the highest scratch response observed in C57 for each pruritogen (compare Figure 2). The peak scratch bouts taken for the calculation were the highest responses observed for each pruritogen and strain in Figure 2. Groups with homogenous means in panels **A** and **B** are marked with the same letters.

Last but not the least, SLIGRL appeared in C57 as the least effective of all tested pruritogens and it produced, at its most potent concentration, 215 ± 60 scratch bouts (10-fold). There was a significant effect of increasing the dose (*F* = 3.7 and *p* = 0.04). Contrary to C3H, SLIGRL, at its most potent concentration (10-fold), produced only 72 ± 13 scratch bouts, which corresponds to a third of the response. In this experiment, only 3 concentrations were tested, however, the influence of dose was apparent (*F* = 5.8, *p* = 0.02, Figure 2G). SLIGRL was the pruritogen where the smallest difference between the two strains became apparent (Figure 3).

Overall, a ranking of all pruritogens according to their potency showed that in both strains, endothelin, chloroquine, and trypsin were the three most potent pruritogens and they produced, across all concentrations, the highest scratch responses (Figures 3A,B). In both strains, endothelin was significantly larger in its effect as compared to any other pruritogen. Then, in both strains, LPA was similar in its effect to chloroquine and trypsin, but also to SLIGRL, histamine, and 5-HT. Then appeared some differences. SLIGRL produced the smallest response in C57, but its effect was comparable to histamine, LPA, 5-HT, and trypsin, but significantly less effective than chloroquine (Figure 3A). In C3H, histamine produced the smallest response, which was different from trypsin and chloroquine, but similar to 5-HT, SLIGRL, and LPA (Figure 3B). When the pruritogens were compared for their maximum effects in both strains, the scratch bouts in C3H reflected between 5% (histamine) and 33% (SLIGRL) of the respective responses observed in C57 (Figure 3C).

**Figure 4:**
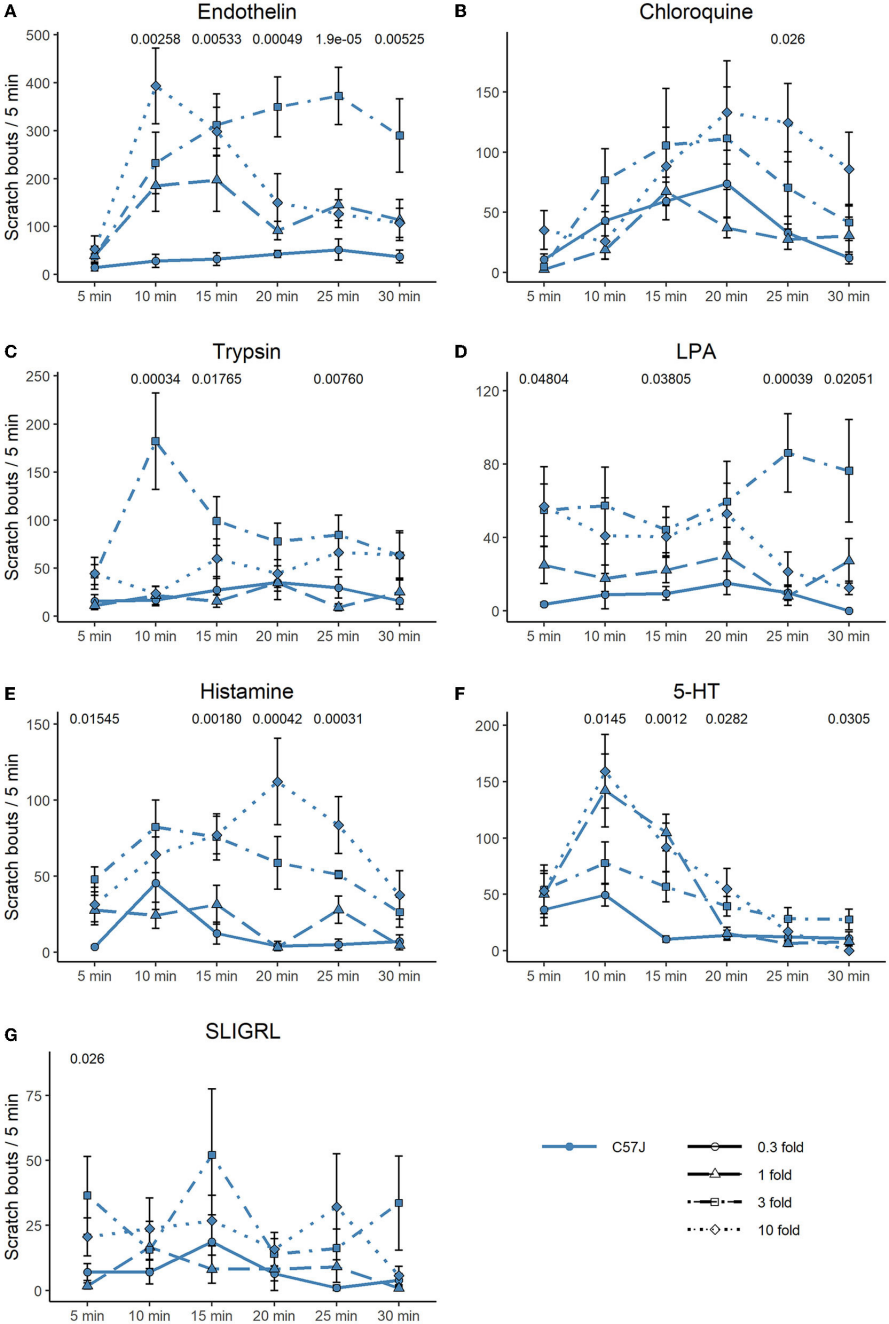
Time-course of scratching bouts in C57. Each data point represents the mean ± SEM of scratch bouts in bins of 5 minutes in response to injection of **(A)** endothelin, **(B)** chloroquine, **(C)** trypsin, **(D)** LPA, **(E)** histamine, **(F)** 5-HT, and **(G)** SLIGRL into the nape of the neck. Four different concentrations were tested. P-values are indicated at the top of each panel and result from a repeated measures ANOVA. The different concentrations are discerned by shape and line type (0.3-fold = circle, solid line; one-fold = triangle, long dashed line; three-fold = square, dot dashed line; 10-fold = diamond, dotted line).

**Figure 5:**
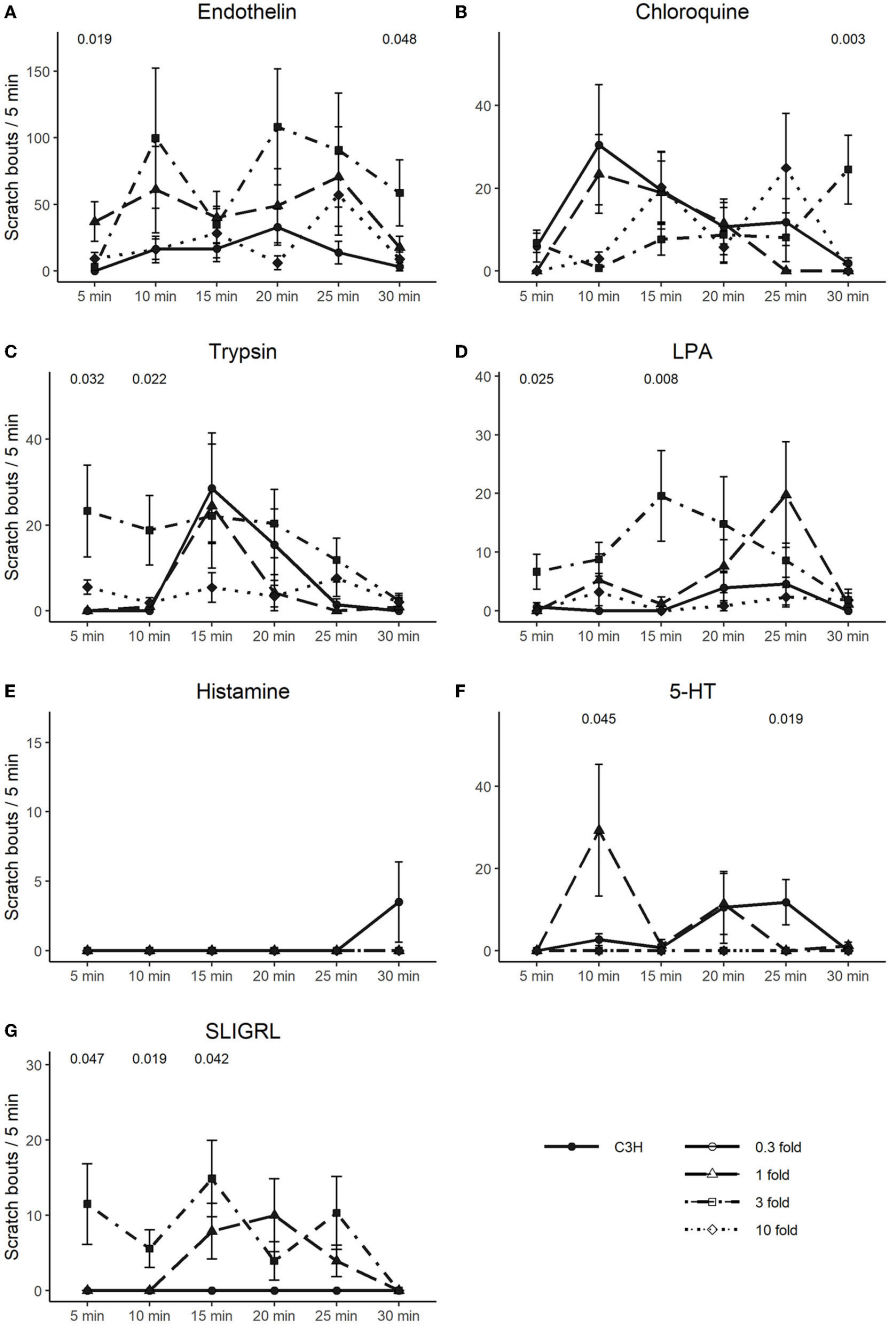
Time-course of scratching bouts in C3H. Each data point represents the mean ± SEM of scratch bouts in bins of 5 min in response to injection of, **(A)** endothelin, **(B)** chloroquine, **(C)** trypsin, **(D)** LPA, **(E)** histamine, **(F)** 5-HT, and **(G)** SLIGRL into the nape of the neck. Four different concentrations were tested. *P*-values are indicated at the top of each panel and result from a repeated measures ANOVA. The different concentrations are discerned by shape and line type (0.3-fold = circle, solid line; one-fold = triangle, long dashed line; three-fold = square, dot dashed line; 10-fold = diamond, dotted line).

In Figures 4, 5, we extended the responses to the pruritogens to analyze the scratch responses in 5 min resolution. We found that endothelin, the most potent pruritogen, produced sustained responses in C57 at all concentrations, except for the highest, where it resulted in a fast decline of the response. Similar observations were true for 5-HT and trypsin, where the highest concentrations produced an immediate scratch response followed by a fast decline. Chloroquine and histamine rather produced a more delayed-onset and long-lasting effect (Figures 4A–G). In C3H, scratch responses were of course lower overall, but the differences between the individual timepoints were also more variable (Figures 5A–G).

## Discussion

In this study, we evaluated the influence of a different genetic background using two inbred mouse strains on the scratch behavior in response to seven commonly known pruritogens. The respective protocol of subsequent application of four different doses, allowed us to identify large strain-dependent differences, and, in C57, we defined peak scratching behavior for three of the seven pruritogens.

The experiments were conducted with an automated experimental setup with limited experimenter bias and with computerized analysis based on defined cutoff frequencies and threshold levels. Inagaki and colleagues were the first to use an automated setup for scratch analysis in mice based on implanted mini-magnets and detection of scratch behavior with coils, but only the method of Kremer is based on keeping the mice in a home-cage-like environment (Inagaki et al., 2002; Kremer et al., 2010). Inagaki validated a software-based method with video-recorded manual scratch counts and used 5–20 Hz, while we used 10–20 Hz. We validated our system using histamine and chloroquine. Our software-based analysis was validated by video recording and manual counting of the number of scratch bouts. Using a frequency of 10–20 Hz to differentiate scratching behavior from non-scratching behavior, we obtained a positive predictive value of 95%, but increasing the frequency range down to 5 Hz did further improve the positive predictive value. The study from Di Mu and colleagues, which revealed the spinobrachial pathway as the major central neural circuit for itch perception, was based on the setup from Inagaki (Mu et al., 2017). Other systems for automated analysis were also introduced more recently. Elliott and colleagues used a method based on acoustic detection of scratching. With this method and validation based on chloroquine-induced scratching, they achieved a reasonable accuracy with 85% sensitivity and a positive predictive value of 75% (Elliott et al., 2017). Escalante and colleagues installed a setup to record 30 min videos of scratching behavior in the home cage from the top, and with the cage lid and food tray removed, but covered with a transparent acrylic glass cover. The scratching frequency, scratching bouts, and grooming time was then calculated offline using custom-based routines (Escalante and Klein, 2020). Last but not the least, Kobayashi et al. introduced a convolutional recurrent neural network-based method to automatically analyze scratching behavior from video material. The network was trained using manually labeled images and then evaluated for accuracy. Sensitivity and positive predictive rates reached as much as 95% and 88%, respectively (Kobayashi et al., 2021).

The pruritogens in this study were selected based on a literature search and their potential to induce an acute itch. To reduce the overall number of mice needed, each mouse received multiple injections throughout the experiment and at least a gap of 48 h were maintained between each treatment (application of the 3Rs principle). In some preliminary tests, we found no sensitization of the mice after multiple subsequent injections of the same substance. Thus, it is assumed that the substances were fully absorbed and metabolized within 48 h and any subsequent scratching behavior was not influenced by prior injections.

In a previous screening study in 30 inbred mouse strains, we assessed diurnal and nocturnal in-cage activities in male and female mice in an automated home cage system (Konig et al., 2020). These measurements were performed in untreated naïve C57 and C3H mice, which were unrelated to the population used for the scratch measurements in the present study. The mice for both studies were raised in the same animal house and under the same housing conditions. C57 and C3H were in groups with a homogenous mean for both the diurnal and nocturnal in-cage ambulatory activity and the diurnal fine movements. Fine movements should be of larger relevance for a relationship with scratch behavior because both are stationary movements. In this respect, the observed differences in scratching behavior between the two strains are not related to different activity levels in the strains and are therefore concluded to be the results of pruritus-specific signaling and related heritable differences (Konig et al., 2020).

In the present study, we pooled the results from both sexes in each group. The principal reason for this approach was the reduction of the overall number of mice needed for the study (application of the 3Rs principle) and previous observations from our unpublished screening study conducted in 21 inbred strains with the respective one-fold pruritogen dose in even numbers of both sexes. In this study, we observed sex differences to be smaller than 12% across all strains. Supplementary Figure 2 displays the sex differences observed in the mentioned study for the two strains, C3H and C57. Apparently, only trypsin and histamine produced different responses in the two sexes (higher scratches in females in response to trypsin and lower in response to histamine). Largely divergent reactions to trypsin and histamine between the sexes were not observed in the present study. Overall, the direct comparison of the pruritogen effects in C57 and C3H is likely not affected by small differences between sexes, because the relative differences between the respective pruritogens across the two strains appeared to be much larger than 12% (see Figure 3C).

Despite our observations of small differences in male and female scratching behavior, the literature database is inconsistent in this matter and does not allow a final conclusion. The first mouse study to address sex differences in pruritogen susceptibility was performed by Green et al. in 2006 and was based on 11 inbred strains and described 23% higher scratching readouts in female individuals following histamine or chloroquine injection in the nape of the neck (Green et al., 2006). In our unpublished screening study in males and females of 21 inbred strains, we identified a smaller difference, however, it is not excluded that sex differences are larger in histamine and chloroquine as compared to other pruritogens or that a few inbred strains drive the sex effect while it is absent in other strains. In this respect, Liang et al. found sex differences in C57 in response to intradermal C48/80, but not due to SLIGRL injection (Liang et al., 2012). In contrast to these studies, Yamaura et al. (2014) tested six pruritogens in ICR outbred mice which included histamine, 4-methylhistamine, 5-HT, C48/80, substance P, and SLIGRL, but only in response to SLIGRL a significant sex difference at a dose of 150 nmol was observed. As far as patients are concerned, we know that the prevalence of chronic pruritus is slightly higher in women, although no significant sex difference was detected in previous studies (Ständer et al., 2010; Kopyciok et al., 2016). In patients, one study found a significant impact of sex on chronic pruritus but only when the lifetime prevalence was taken into account (Matterne et al., 2011).

In previous studies, C57BL/6J and Balb/cJ mice were the preferred inbred strains for the quantification of scratch behavior and the research on itch-related molecular mechanisms. Both strains have more pronounced scratching responses than C3H (Green et al., 2006). Previous studies found a relationship between pruritogen dose and acute scratch response, but a comparison of dose-relationships and maximum responses for inbred strains with opposite phenotypes has so far not been attempted and they would allow the conclusion of a general resistance or low sensitivity of specific strains to develop itch.

In the literature, full dose relationships and peak scratching responses are available for chloroquine in 11 strains, provided by Green et al. Information is also available for trypsin in a C57BL/6-related wildtype strain, evoking a scratch maximum at 300 μg which was not further increased by the injection of 500 μg (Costa et al., 2008). In our hands, trypsin was among the three most potent pruritogens in both strains, and the dose relationships in both strains revealed that the peak responses were not yet reached with the maximum dose applied in our study. With respect to histamine, chloroquine, and 5-HT, we assume that the peak responses correspond to the respective threefold concentrations, as these seemed to produce the maximum response possible in the C57 strain. The respective tenfold concentrations produced visibly reduced, but not significantly smaller scratch bout responses. In C3H in contrast, we did not encounter a relevant dose-response relationship for chloroquine or histamine, because it would have been required to further increase the dose with the risk of systemic side effects and C3H responded with a maximum response of 27 or 5% of the respective peak response observed in C57. Similar to endothelin, trypsin, LPA, and SLIGRL, the maximum doses were either as high as 10F or even higher. Therefore, it would have been required to increase the doses further to obtain the peak scratching response. Nevertheless, their potency in C57 was several folds higher than in the C3H strain for all tested concentrations.

SLIGRL appeared to be the most potent pruritogen in C3H relative to C57 - at least it evoked the largest scratch response magnitude observed relative to C57 (33%, Figure 3C). Previous studies showed that the PAR-2- agonist peptide SLIGRL affects scratch response in a dose-dependent manner (Akiyama et al., 2012a; Yamaura et al., 2014; Coavoy-Sánchez et al., 2016). All studies, which were based on C57 mice or outbred mice, showed a robust scratch response to SLIGRL, whereas, in our study, this pruritogen produced a comparably weak response in C57. C3H mice seemed to be more sensitive to SLIGRL as compared to other pruritogens when the respective difference in scratch bouts was related to C57. In C57, medium (100 nmol) or lower doses (76 nmol) revealed an up to five times higher number of scratches in published studies as compared to our findings shown in the present study (Akiyama et al., 2012b; Coavoy-Sánchez et al., 2016; Ma et al., 2020). So far, lower scratch responses to SLIGRL were previously only observed in Sprague–Dawley rats (Klein et al., 2011).

Endothelin was the most potent pruritogen in both strains. Endothelin is a 21-amino acid peptide that exerts sensory effects via the endothelin-A (ET-A) and -B receptors expressed in endothelial and immune cells and in sensory neurons. Although the difference between both strains was almost five-fold, the effect of endothelin was more potent than any other pruritogen. *In vitro* and *ex vivo* experiments provided evidence, that endothelin activates pruritogen-sensitive and nociceptive neurons and triggers both itch and pain signals or simultaneous sensations of itch and pain (Akiyama et al., 2015). So far, the endothelin-induced scratching behavior was shown to be attenuated by ET-A and TRPA1 receptor antagonism, and most endothelin-sensitive neurons additionally respond to histamine, chloroquine, and SLIGRL and are sensitive to bombesin (Akiyama et al., 2014).

The gastrin-releasing peptide receptor is a member of the bombesin subfamily of GPCRs and its expression in the lamina I of the dorsal spinal cord marks a specific pathway for itch which does not affect the transmission of nociceptive signals (Sun and Chen, 2007; Sun et al., 2009). Similarly, the neurotransmitter b-type natriuretic peptide (BNP) is expressed in a subset of primary afferent neurons which are specifically transmitting itch, and mice with ablated genes lack the responses to pruritogens including histamine, chloroquine, endothelin 1, serotonin, compound 48/80 and PAR2 agonist SLIGRL-NH2 (Mishra and Hoon, 2013). Meanwhile, potential targets and possible strategies against BNP signaling are considered to modulate chronic itch (Meng et al., 2020). Recent studies brought also evidence that the opioidergic system is a potent modulator of itch transmission in general and that increased μ-opioid activity and decreased κ-opioid activity contribute to itching (Wikström et al., 2005; Melo et al., 2018).

Genes with modulatory effect on the signal transmission in itch-dedicated pathways as the cause of the observed differences between the two strains were not yet addressed, but a genetic difference that modulates itch transmission at a junction point may be one possible explanation for the observed general resistance to a broad range of pruritogens in C3H. Other possible mechanisms may be differences in expression levels of relevant proteins. A negative correlation between chloroquine-induced itch and thermal pain (49°C tail immersion) was proposed based on a selection of 11 inbred strains and failed only for Balb/cJ (Green et al., 2006). A study by Mogil on the heritability of nociception showed that C3H had a higher sensitivity in several nociceptive assays where they behaved opposite to C57. The relevant nociceptive assays were the 49°C tail withdrawal and the 53°C hot-plate tests of thermal sensitivity, the carrageenan thermal hypersensitivity assay, and the abdominal constriction tests (based on acetic acid and magnesium sulfate), but not the Hargreaves test (Mogil et al., 1999). Our observations confirmed that the resistance to itch extends from histamine and chloroquine to all other pruritogens, thus affecting the entire itch pathway. The group of C3H-like strains in these nociceptive assays (Mogil et al., 1999) are hence expected to share the same low sensitivity to itch (Green et al., 2006).

In general, the comparison of dose ranges is the appropriate tool to identify strains as resistant. Whether the sensitivity to these pruritogens generalizes to models of chronic pruritus needs to be discovered. The strains with robust opposite phenotypes in this trait are helpful models for the discovery of genetic factors that modulate the transmission of pruritus or convey itch variability. Once genetic factors for itch variability genes in the mouse have been sighted, translational genetic association approaches in humans are possible. They may ultimately lead to the identification of homologous genes and therapeutic options for the alleviation of pruritus in a clinical context.

## Data availability statement

The raw data supporting the conclusions of this article will be made available by the authors, without undue reservation.

## Ethics statement

All research and animal care procedures were reviewed by the Ethics Committee of the Friedrich-Alexander-University Erlangen-Nürnberg (Regierung von Unterfranken) under the file number 55.2 DMS-2532-2-240. Experiments involving live animals were conducted in accordance with the International Association for the Study of Pain Guidelines for the Use of Animals in Research.

## Author contributions

Conception or design of the work: KZ and AK. Data collection: YZ and CK. Data analysis and drafting the Figures: YZ, NR, and CK. Drafting the article: YZ and KZ. All authors critically revised and approved the article.

## Funding

YZ was supported by a fellowship from Zhengzhou University First Affiliated Hospital, No. 1 Jianshe East Road, Zhengzhou City, Henan Province, People's Republic of China and from the German Academic Exchange Service (DAAD) via the Department of International Affairs, University of Erlangen-Nürnberg. The project was funded by the Deutsche Forschungsgemeinschaft (DFG, German Research Foundation) - project numbers ZI1172/4-4 to KZ, KR3618/3-1, and KR3618/3-2 to AK.

## Conflict of interest

The authors declare that the research was conducted in the absence of any commercial or financial relationships that could be construed as a potential conflict of interest.

## Publisher's note

All claims expressed in this article are solely those of the authors and do not necessarily represent those of their affiliated organizations, or those of the publisher, the editors and the reviewers. Any product that may be evaluated in this article, or claim that may be made by its manufacturer, is not guaranteed or endorsed by the publisher.
