# Supplementary material for: Generalized resistance to pruritogen-induced scratching in the C3H/HeJ strain
Source: Front Mol Neurosci. 2022 Oct 5;15:934564. doi: 10.3389/fnmol.2022.934564 (PMC9581333; doi:10.3389/fnmol.2022.934564)
Supplement: Supplementary file 1 [file Data_Sheet_1.pdf]

# Supplementary material

## Figure S1

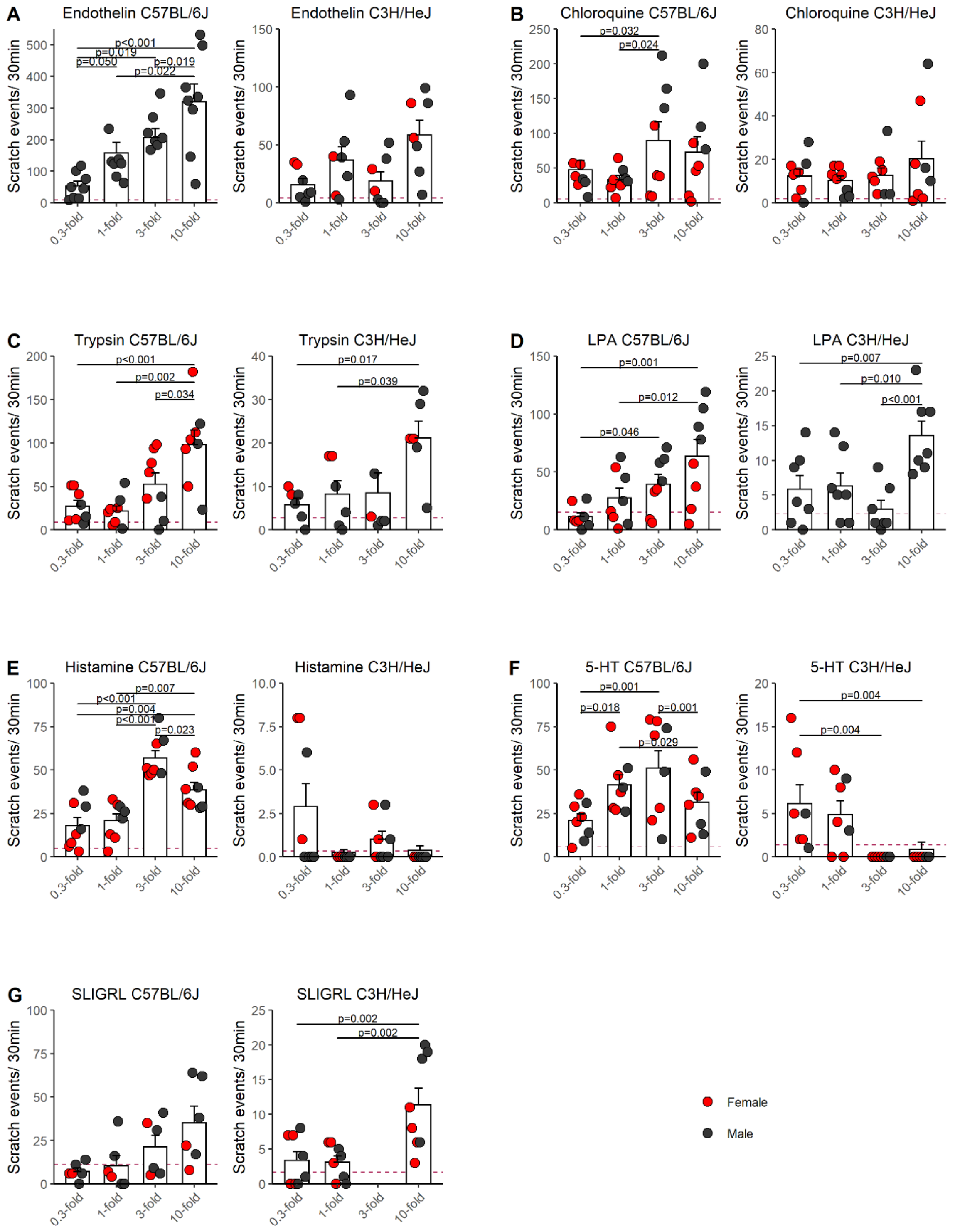

**Dose–response relationships of scratching behavior in C57 and C3H mice for seven pruritogens analyzed as scratch events.** Columns represent means ± SEM of scratch events in 30 min following an injection of, **A.** endothelin (0.3 - 10 µM; C57 n = 8 males; C3H n = 7, 2 females, 5 males), **B.** chloroquine (1.2 – 40 mM; C57 n = 6, 4 females, 2 males; C3H n = 8, 3 females, 5 males), **C.** trypsin (3.03 – 101 U/µl; C57 n = 8, 5 females, 3 males; C3H n = 6, 2 females, 4 males ), **D.** LPA (1.2 – 40 mM; C57 n = 8, 4 females, 4 males; C3H n = 7, 7 males), **E.** histamine (26.7 – 890 mM; C57 n = 6, 4 females, 2 males; C3H n = 8, 3 females, 5 males), **F.** 5-HT (0.3 – 10 mM; C57 n = 8, 5 females, 3 males; C3H n = 7, 5 females, 2 males), **G.** SLIGRL (0.6 – 20 mM; C57 n = 7, 2 females, 4 males; C3H n = 8, 4 females, 4 males) into the nape of the neck. In C57, some of the pruritogens exhibited linear dose-response relationships (endothelin, trypsin, LPA and SLIGR) and, contrast to the scratch bouts-based analysis inverted-U-shaped curves (5-HT and histamine). All pruritogens were several-fold more potent in C57 as compared to C3H. P-values result from a repeated measures ANOVA with LSD PostHoc Test. Data points from females are in red, males in black.

# Figure S2

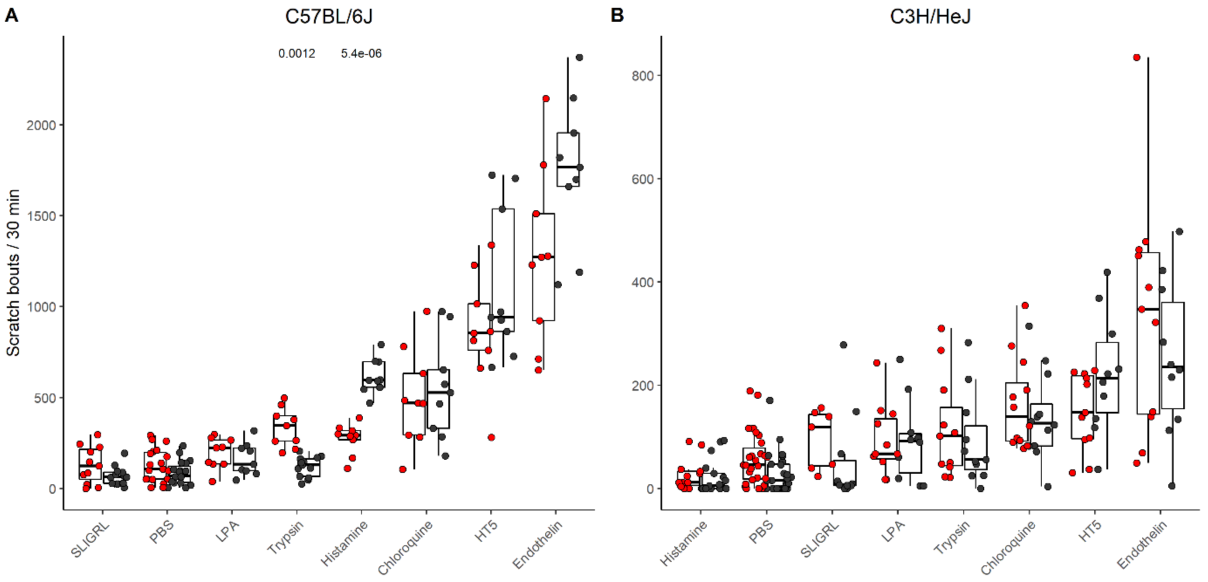


**Sex-difference in pruritogen-induced scratching behavior in C57 and C3H.** Boxes represent means ± SD of scratch bouts in 30 min following an injection of the respective indicated pruritogen. The whiskers represent minimum and maximum, boxes the quartiles and the horizontal lines the mean. An outlier analysis was performed using the 2.2-fold of the interquartile range. PBS signifies phosphate-buffered saline and represents the scratch response to the solvent control. All pruritogens were applied in the respective 1-fold concentrations (see table 2). Data are from a 21 inbred strain screening study. The PBS-induced scratch values were then used to interpolate the control levels for each pruritogen in main Figure 2.

# Figure S3

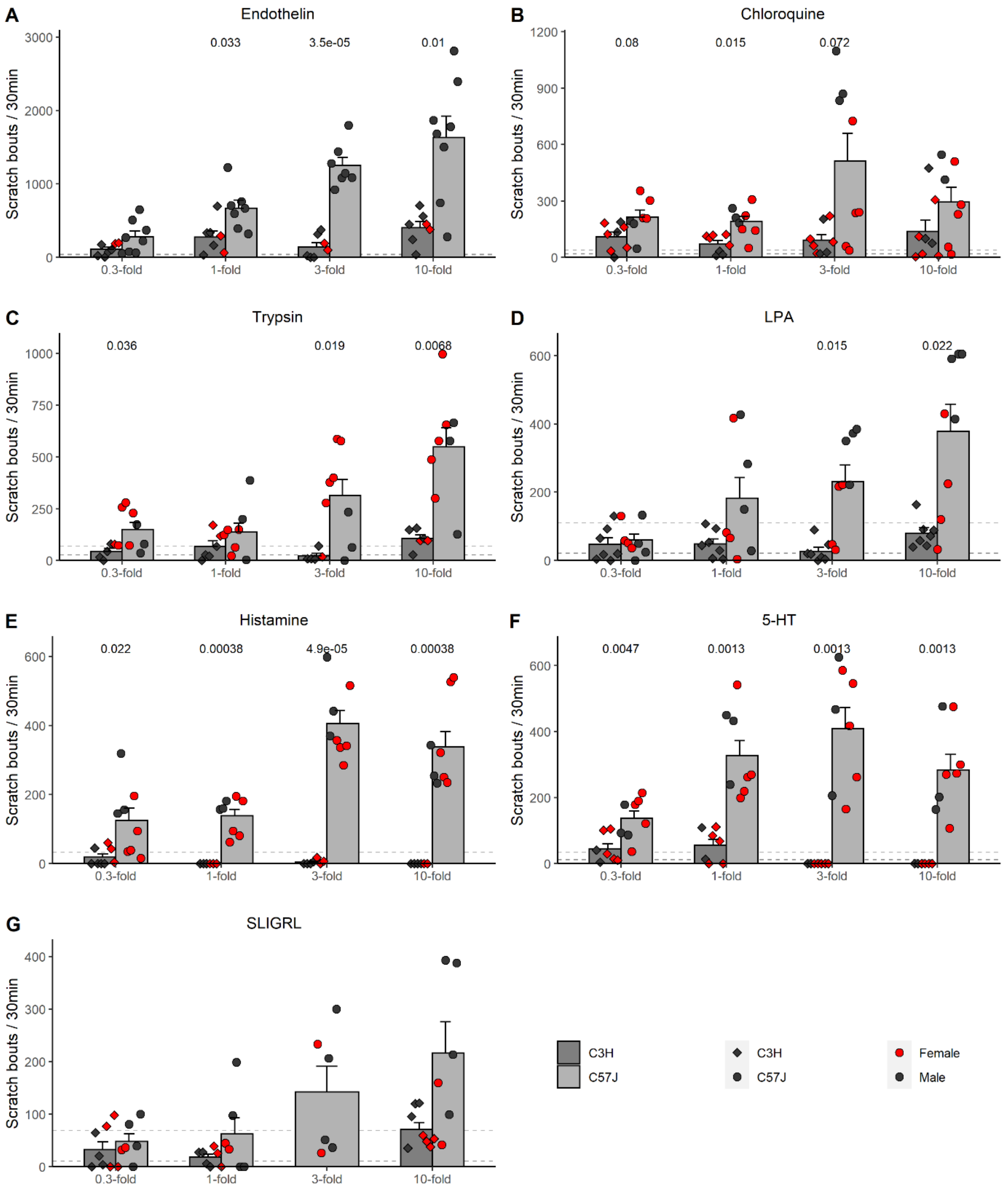


**Comparison of scratch responses observed in C57 and C3H in response to seven pruritogens.** Columns represent means ± SEM of scratch bouts in 30 min following an injection of, **A.** endothelin, **B.** chloroquine, **C.** trypsin, **D.** LPA, **E.** histamine, **F.** 5-HT and **G.** SLIGRL into the nape of the neck. All pruritogens were several-fold more potent in C57 as

compared to C3H. The dashed lines indicate the mean response of an interpolated PBS control from a different strain-specific animal cohort (see Figure S2). The PBS control was interpolated based on the 1-fold concentration of each pruritogen. The values inserted at the top of columns are P-values and they result from Student's t-test with a Holm-Bonferroni-adjustment. Not normal distributed data (Shapiro-Wilk test) was compared by using Wilcoxon rank-sum test. C57 are represented in light grey columns (mean) and circles (data points) and C3H as dark grey columns (mean) and diamonds (data points). Data points from females are in red, males in black.
